# Neuromuscular Blockade Monitoring: Having It but Knowing When Not to Trust It

**DOI:** 10.7759/cureus.45438

**Published:** 2023-09-18

**Authors:** Sara Salvador, Rita Frada, Matilde Campos, Simão Esteves

**Affiliations:** 1 Anesthesiology, Centro Hospitalar Universitário de Santo António, Porto, PRT

**Keywords:** neuromuscular block monitoring, train-of-four (ratio), neuromuscular blocking agents, general anesthesia, butyrylcholinesterase deficit

## Abstract

Butyrylcholinesterase (BChE) is an enzyme involved in the degradation of depolarizing and non-depolarizing neuromuscular blocking agents (NMBA), such as succinylcholine and mivacurium, respectively. Its deficiency is inherited or acquired, and results in paralysis of skeletal muscles after NMBA administration. We report a case of a 32-year-old pregnant woman proposed for cesarean section. General anesthesia (GA) was induced using propofol and succinylcholine. The surgical procedure was uneventful but after 40 minutes, there was no reversal of neuromuscular block (NMB). Other differential diagnoses were excluded and a deficit of BChe was assumed. When the train-of-four ratio (TOFr) achieved 40%, neostigmine/atropine led to the slow recovery of NMB up to TOFr 88%. The patient was extubated, but ventilation proved ineffective, so GA was induced and the patient was reintubated. A new measurement found a TOFr of 60%. Sedation and ventilatory support were maintained until the complete reversal of NMB (4 hours after succinylcholine). Prolonged block is a rare but serious complication of the use of succinylcholine in patients with BChE deficiency. This report not only highlights the importance of intraoperative NMB monitoring in homozygotic patients for atypical cholinesterase but also raises awareness for its careful interpretation.

## Introduction

Butyrylcholinesterase (BchE) also known as pseudocholinesterase, plasma cholinesterase, or serum cholinesterase is an enzyme synthesized in the liver that circulates in plasma. Although it has no clear physiologic function, it is involved in the degradation of depolarizing and non-depolarizing neuromuscular blocking agents (NMBA), such as succinylcholine and mivacurium, respectively. The action of succinylcholine is short in onset (45 seconds) and is used to facilitate tracheal intubation on rapid sequence induction of anesthesia. It lasts nine to 12 minutes but BChe deficiency is either inherited or acquired and results in paralysis of skeletal muscles and prolonged apnea after the administration of these agents [1].

Congenital BchE deficiency is an autosomal recessive trait that affects approximately one in 2000 to 5000 individuals. Several genetic variations have been identified with different diagnostic and clinical effects [2].

## Case presentation

We report a case of a 32-year-old pregnant woman (102 Kg; body mass index 37.9 Kg/m^2^) electively admitted for labor induction. She had a history of asymptomatic Leiden factor V mutation and gestational diabetes and took enoxaparin (40 mg) and aspirin (100 mg) daily during the pregnancy. She had a previous spontaneous abortion requiring uterine curettage without any anesthetic complication reported or NMBA administration. Preoperative laboratory tests and electrocardiography were normal. The patient denied any allergies. In addition, there was no family history of problems associated with anesthesia. This case report was published with the written consent of the patient.

**Figure 1:**
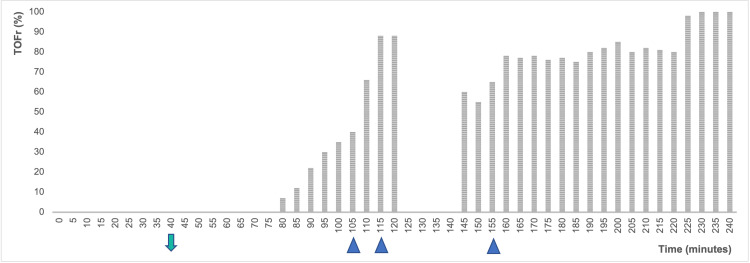
Timeline of TOF ratio throughout the event 0 - General anesthesia induction; Down arrow - end of surgery; Up arrow - intravenous administration of atropine and neostigmine (see doses on text)

Due to failure to induce labor, she was proposed for a low cervical cesarean section at 40 weeks. As an epidural catheter was already in place, a top-up with 0.75% ropivacaine (10 mL) was administered. After the sensitive block reached T4, the surgery began. However, the patient complained of pain at the surgical incision, so GA was induced with rapid-sequential induction using propofol (200 mg) and succinylcholine (100 mg). No fasciculations were observed at this point. Intubation with videolaryngoscope (C-MAC®) and endotracheal tube succeeded without complications. Besides standard American Society of Anesthesiology (ASA) monitoring, processed electroencephalogram (BIS®) and quantitative neuromuscular monitoring (Mechanosensor®) were added after GA induction. Anesthetic maintenance was attained with propofol infusion according to BIS monitoring. The surgical procedure was uneventful. As seen in Figure 1, after 40 minutes of succinylcholine administration, there was no reversal of NMB (no TOF (train-of-four) responses and absent post-tetanic count).

Other differential diagnoses, such as malfunction of the device (monitor, cable, and sensor), electrolyte alterations, hypoglycemia, or hypothermia were excluded. Nearly two hours after succinylcholine administration, the motor block reversal demonstrated a fading pattern (phase II). When the train-of-four ratio (TOFr) was 40%, neostigmine (1.5 + 1.5 mg) and atropine (0.5 + 0.5 mg) were given and a slow recovery of blockade up to TOFr of 88% was observed for 10 minutes. A respiratory regular effort was detected with a tidal volume of 4 mL/Kg, so propofol infusion was paused. As the patient was obeying commands, she was extubated. However, in the following 20 minutes, she reported dyspnea and presented signs of respiratory distress. GA was again induced with propofol (200 mg) and the airway was secured with an endotracheal tube (similar to the first intubation). Succinylcholine was not given at this point. A new NMB assessment revealed a TOFr of 60%. At this stage, more neostigmine (2.5 mg) and atropine (1 mg) were given without any effect. Sedation and ventilatory support were maintained until complete and sustained reversal of NMB (TOFr 100%) 4 hours after succinylcholine administration. The patient remained in the theater until extubation and completed a prolonged recovery in the post-anesthetic care unit afterward. From the postoperative study, the patient had lower than normal plasma cholinesterase dosing (1224 u/L - reference values for age and pregnancy between 3650 and 9120 u/L) and dibucaine number 37% (normal range >75%). The lab results six weeks after surgery showed a plasma cholinesterase dosing of 3767 u/L and dibucaine number 51%.

## Discussion

Delayed emergence is defined as the failure to regain consciousness after GA. It is a challenge every anesthesiologist should be prepared to manage. Effects of hypnotics and other drugs (e.g. opioids and benzodiazepines), hypoxemia, hypercarbia, hypoglycemia, and new neurologic deficits should be ruled out. Before considering BChe deficit, other causes for intraoperative prolonged NMB need to be accessed such as electrolytic changes (hypokalemia, hypocalcemia, and hypermagnesemia) and hypothermia [2].

Succinylcholine consists of two acetylcholine molecules linked, which compete with acetylcholine for the cholinergic receptors and depolarize the endplate. NMB with succinylcholine changes from phase I (depolarizing) to phase II (nondepolarizing or desensitizing) block. Initially, the depolarizing activity predominates. However, with increasing dose or time nondepolarizing occurs gradually. Commonly, there is no need to reverse the succinylcholine effect, as time allows for spontaneous recovery. During a phase I block, neostigmine will directly potentiate the block by inhibiting BchE and prolonging the presence of nonhydrolyzed succinylcholine. Thus, neostigmine may be used to antagonize succinylcholine block only when there is clinical evidence of pure phase II block and train-of-four fade has been documented [3]. European guidelines [4] on NMB management recommend a dose of neostigmine of 40 ug Kg1 and advanced spontaneous recovery (TOFr >20%) with an expected reversal within 10 to 15 min.

In patients with BChe deficit, publications have experienced mixed results with the use of anticholinesterase showing complete or partial reversal and block potentiation. This likely depends on the plasmatic levels of succinylcholine and the simultaneous presence of phase I and phase II blocks [5-7].

After neostigmine administration, the patient presented with a slow improvement of TOFr up to 88%, which was interpreted as a pure non-depolarizing block. But the extubation was unsuccessful and the following evaluation revealed a TOFr of 60%. The re-establishment of paralysis after neostigmine administration has been described previously in patients with BChe deficit [5-8]. In one of the cases from Savarese, an absolute height of the TOFr and single twitch occurred over the subsequent 90 minutes of neostigmine administration without change in the TOFr per se and an improvement in the patient’s clinical condition [6]. Nonetheless, in this case, such change was not noticeable.

The sequence of events suggests that a mixed block was present and the fade on the TOF might not reflect a pure nondepolarizing block. In this case, it is possible that repeated doses of anticholinesterases delay recovery from NMB by decreasing the already low plasma cholinesterase activity, and probably the second administration of neostigmine could have been avoided. Due to the unpredictability of the neostigmine effect, some authors do not recommend its use in patients with a BChE deficit [9].

Furthermore, this case also emphasizes that residual NMB cannot be discarded by clinical tests, as the patient was obeying commands and maintaining a five-second head lift but was still unable to keep adequate ventilation. In healthy volunteers, it is known that the ability to lift the head or hold a firm grip is not reliable as it can be performed when TOFr is above 50% [10-12]. Quantitative monitoring of NMB is crucial and should be evaluated whenever muscle relaxants are used [13]. The monitoring gains further importance in patients with a BChE deficit as shown in previously reported cases, where other NMBA was used [8] or the diagnosis of the disease was delayed [14]. In this case, although a quick assessment was carried out (standard and BIS monitoring, neurological exam, arterial blood gas, electrolyte, glucose, and temperature), the clinical findings and NMB monitoring easily pointed to residual paralysis as the cause of the delayed emergency.

Although the most common genetic variant is the K-variant with a frequency of about 20% in Caucasians, the atypical variant has the most clinical significance. The silent variants with no enzyme activity are very rare [2].

Regarding diagnosis, there are several tests that can be used. One of the most common is the dibucaine inhibition test. Dibucaine is a local anesthetic that inhibits 80% of BChE activity in standard people. Heterozygotes for the atypical variant will inhibit BChE enzyme activity by 40-60% and homozygotes by 20%. Sensitivity to both succinylcholine and mivacurium can prolong the NMB by up to eight hours in homozygotic and up to two hours in heterozygotic patients. Acquired reduced BChE activity may also occur in patients with chronic liver disease, renal and heart failure, malnutrition, malignancies, severe burn injuries, or because of drug interactions (oral contraceptives, cyclophosphamide). A physiological decrease in BChe activity of 20% in the first trimester of pregnancy is maintained until delivery [1,2].

The dibucaine number, plasma cholinesterase, and time of prolonged NMB dosing suggested a homozygotic atypical cholinesterase, however, the results six weeks after surgery showed a plasma cholinesterase dosing of 3767 u/L (earlier 1224 u/L) and dibucaine number 51% (earlier 37%). Although these tests are helpful in defining a grade of deficiency, they cannot identify pseudocholinesterase genotypes. Both mother and child await genetic testing.

## Conclusions

Prolonged block is a rare but serious complication of the use of succinylcholine in patients with BChE deficiency. This report highlights the importance of intraoperative NMB monitoring in homozygotic patients for atypical cholinesterase, but also raises awareness of its interpretation, as the presence of fade on TOFr does not translate a pure phase II block or guarantees a successful antagonism by anticholinesterases. The block could be present even after apparent decurarization.
